# The Function of the Conserved Regulatory Element within the Second Intron of the Mammalian *Csf1r* Locus

**DOI:** 10.1371/journal.pone.0054935

**Published:** 2013-01-31

**Authors:** Kristin A. Sauter, M. Amine Bouhlel, Julie O’Neal, David P. Sester, Hiromi Tagoh, Richard M. Ingram, Clare Pridans, Constanze Bonifer, David A. Hume

**Affiliations:** 1 Developmental Biology, The Roslin Institute, Royal (Dick) School of Veterinary Studies, University of Edinburgh, Roslin, United Kingdom; 2 Section of Experimental Haematology, Leeds Institute of Molecular Medicine, University of Leeds, St James’s University Hospital, Leeds, United Kingdom; 3 School of Cancer Sciences, Institute of Biomedical Research, College of Medical and Dental Sciences, University of Birmingham, Birmingham, United Kingdom; Center of Ophtalmology, Germany

## Abstract

The gene encoding the receptor for macrophage colony-stimulating factor (CSF-1R) is expressed exclusively in cells of the myeloid lineages as well as trophoblasts. A conserved element in the second intron, Fms-Intronic Regulatory Element (FIRE), is essential for macrophage-specific transcription of the gene. However, the molecular details of how FIRE activity is regulated and how it impacts the *Csf1r* promoter have not been characterised. Here we show that agents that down-modulate *Csf1r* mRNA transcription regulated promoter activity altered the occupancy of key FIRE cis-acting elements including RUNX1, AP1, and Sp1 binding sites. We demonstrate that FIRE acts as an anti-sense promoter in macrophages and reversal of FIRE orientation within its native context greatly reduced enhancer activity in macrophages. Mutation of transcription initiation sites within FIRE also reduced transcription. These results demonstrate that FIRE is an orientation-specific transcribed enhancer element.

## Introduction

Macrophage colony-stimulating factor (CSF-1) controls the proliferation, differentiation and survival of cells of the mononuclear phagocyte lineage [1], [2]. CSF-1 mediates its actions by binding to the CSF-1 receptor (CSF-1R), a type III receptor protein tyrosine kinase. Expression of the *Csf1r* gene is switched on early in myeloid lineage commitment, and expression levels increase as immature myeloid cells differentiate into mature macrophages [3]. The *Csf1r* transcript in macrophages is expressed from a purine-rich promoter that lacks a TATA box and other classical elements that specify the transcriptional start site, which are characteristics shared with many other myeloid promoters [4]–[6].

The proximal promoter of *Csf1r* is not sufficient to generate maximal expression but requires the enhancer activity of a highly-conserved ∼330 bp sequence in the second intron downstream of the macrophage promoter, which we named the Fms-Intronic Regulatory Element (FIRE; [7], [8]). This element is functionally conserved between human and mouse [9] and the chicken *Csf1r* locus also contains a conserved element in an equivalent location within the first intron [10]. Deletion of FIRE from a GFP reporter construct containing the promoter and downstream intron 2 sequence abolishes GFP expression in transgenic mice [8]. FIRE contains multiple binding sites for myeloid transcription factors commonly found in myeloid promoters including PU.1, RUNX1, SP1/3, and AP-1 and lies within open chromatin in macrophages. FIRE was shown to possess anti-sense promoter activity in transient transfections of a macrophage cell line and subsequently, two anti-sense transcription start sites were mapped within FIRE [3]. Antisense promoter activity of FIRE was activated in B cells by PAX5, and was associated with repression of *Csf1r* expression [11].

Numerous genome-scale studies now indicate that promoter activity associated with enhancers is widespread, producing what have been called eRNA [12]–[14]. The function of these transcripts appears to be highly variable. In the case of the beta-globin locus, there is some evidence that transcription initiated from an upstream enhancer has an essential function in the generation of a chromatin loop that link enhancer and promoter [15] whereas other eRNAs appear to be bystander products of enhancer activity with as yet unknown functions [16]. In this paper, we present evidence that FIRE is an anti-sense RNA Polymerase II promoter in macrophages and is involved in fine-tuning *Csf1r* expression in response to stimuli. We provide evidence that the enhancer activity of FIRE in its native context is orientation-dependent and requires transcription initiation sites.

## Results

### Anti-sense FIRE Promoter Activity is Increased in the Presence of Stimuli that Down-regulate *Csf1r* Expression

**Figure 1:**
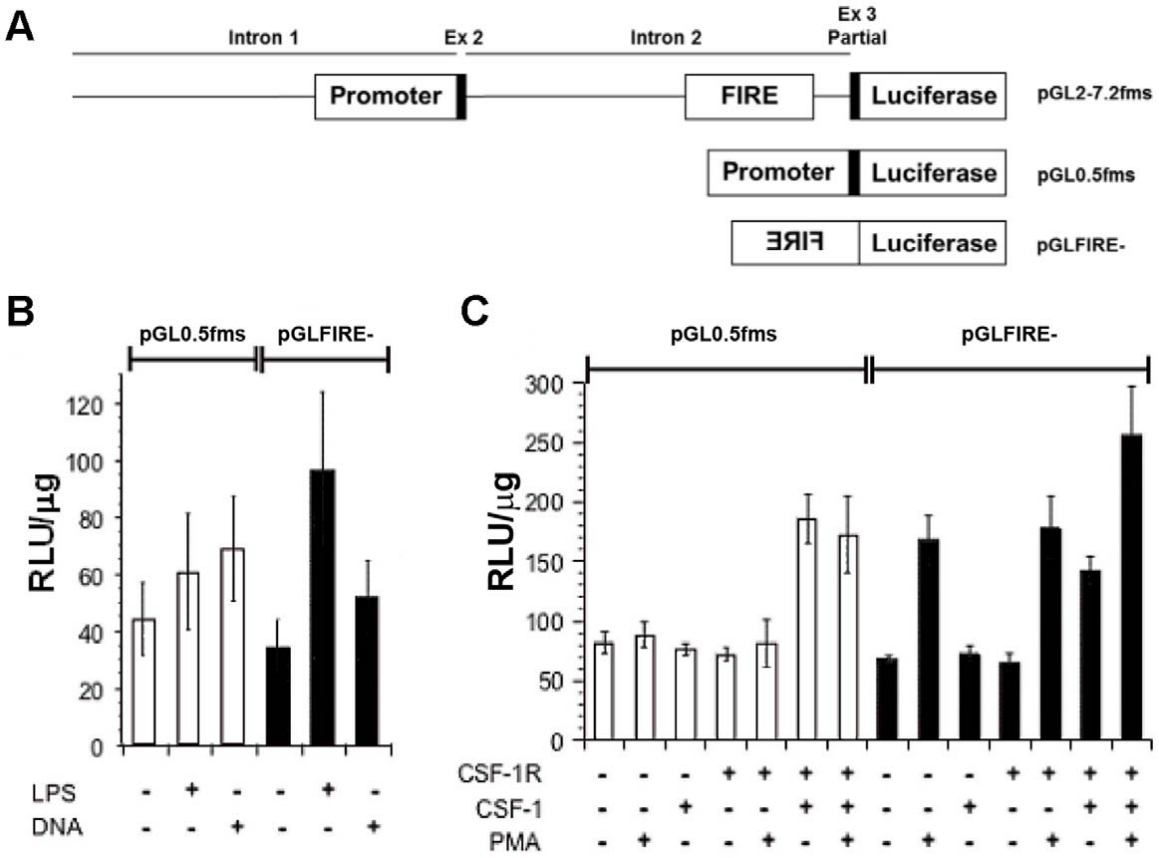
The FIRE region acts as an inducible promoter. (**A**) Schematic of FIRE constructs: the entire *Csf1r* regulatory region plasmid (pGL-7.2 fms), for reference, the *Csf1r* promoter cloned into the pGL2B vector upstream of a luciferase reporter (pGL0.5 fms), FIRE in reverse orientation was cloned into the pGL2B vector upstream of a luciferase reporter (pGLFIRE-). (**B**) RAW264.7 cells were transfected with pGL0.5 fms or pGLFIRE-. Transfected cells were treated (+) with either LPS or bacterial DNA or left untreated (−) for 8 hours before assay of luciferase activity. (**C**) Naïve RAW264.7 cells (CSF-1R −) or RAW264.7 cells stable transfected with a CSF-1R expression plasmid (CSF-1R +) were transfected with reporter constructs as above and treated (+) with CSF-1 for 20 hours, PMA for 8 hours, or both CSF-1 & PMA or left untreated (−) before assay of luciferase activity. Columns in B & C represent the mean RLU/µg protein and error bars the SEM of three independent assays which each showed the same pattern.

FIRE was shown previously to exhibit directional promoter activity in transient and stable transfection assays of the macrophage cell line RAW264.7 [7], suggesting a regulatory function of anti-sense transcripts initiating within FIRE. We therefore examined whether treatments that regulate *Csf1r* transcription acted through FIRE and tested three such stimuli, toll-like receptor agonists, CSF-1 and phorbol esters (PMA) [21] all of which impact *Csf1r* expression. In transient transfections using a construct carrying only the sense promoter, reporter gene expression was unaffected by TLR agonists (LPS, bacterial DNA) whereas FIRE anti-sense promoter activity was induced 3.2 fold with LPS stimulation and 1.7 fold, (*p*<0.05) by bacterial DNA treatment (**Figure 1A & B**). Since RAW264.7 cells express relatively low levels of receptor on the cell surface and do not respond to CSF-1 treatment [22], we generated a stable cell line over-expressing CSF-1R. This cell line was transfected with the *Csf1r* promoter or the FIRE enhancer cloned in the antisense orientation, and then treated with CSF-1, PMA, or both. CSF-1 actually induced the promoter as it has been reported to do in fibroblasts expressing CSF-1R [23]. Both CSF-1 and PMA induced FIRE promoter activity (**Figure 1A & C**). Together, these data demonstrate that the anti-sense promoter activity of FIRE is enhanced in the presence of stimuli that normally downregulate *Csf1r* expression.

### *Csf1r* Primary and Antisense Transcripts Increase in Response to CSF-1 Deprivation While LPS & CSF-1 Cause a Rapid Down-regulation

**Figure 2:**
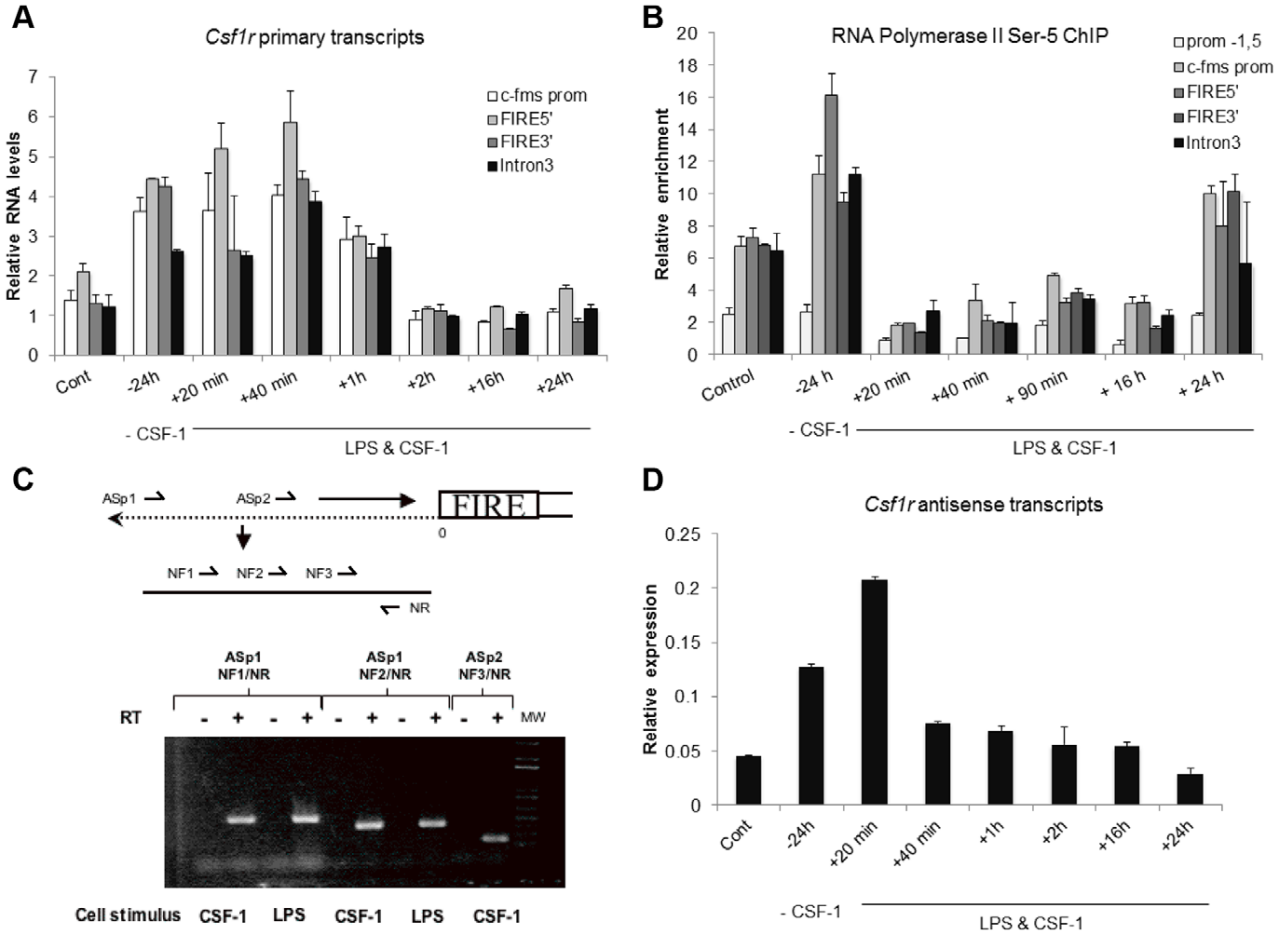
Effect of CSF-1 and LPS on *Csf1r* primary and antisense transcripts. (**A**) Macrophages were differentiated from mouse bone marrow under the influence of CSF-1. Cells were starved of CSF-1 for 24 hours, and then re-stimulated with a combination of CSF-1 and LPS. Primary RNA levels containing intronic sequences were measured by Real-Time PCR assays using primers downstream of the promoter, FIRE or intron 3. Normalisation was performed using rRNA-specific primers. Error bars represent the mean value of triplicate PCRs. (**B**) Cells and treatments were identical to (A). ChIP assays measuring the recruitment of Serine 5 phosphorylated RNA Pol II using primers covering the indicated cis-regulatory elements. Prom – 1.5 kb refers to a region upstream of the transcription start site which served as an internal negative control [29]. Values were normalised against an internal negative control as described in materials and methods. Measurements are representative of at least two independent experiments and error bars represent the mean value of three different measurements. (**C**) Primers were produced that contain sequence from the positive strand of *Csf1r* upstream of the FIRE region within intron 2 (Asp1 or Asp2). These primers prime negative strand transcripts by reverse transcriptase reaction (+). DNAse treated RNA was used for the reaction and as a control RNA was primed in the absence of reverse transcriptase (−). cDNA products were detected by PCR with nested forward (NF1, NF2, or NF3) and reverse (NR) primers. (**D**) Cells and treatments were identical to (A). Antisense RNA-expression was assayed by real-time quantitative PCR exactly as described in [20].

FIRE is located in open chromatin in mouse and human macrophages [24] and tagged with H3K4Me3 suggesting an active promoter [25]. It has been previously reported that *Csf1r* mRNA expression is auto-regulated by its ligand. Growth factor withdrawal up-regulates *Csf1r* expression while inflammatory stimuli, such as LPS, down regulate expression. Both our own data, and others [26], [27] indicate that *Csf1r* down-modulation in macrophages by LPS is associated with diminished production of transcripts as detected in nuclear run-on transcription assays. To confirm that this regulation takes place at the level of transcription initiation, we measured primary transcripts using different primers across the transcribed region. These measurements were combined with chromatin immunoprecipitation (ChIP) experiments testing for the recruitment of the initiating Serine 5 phosphorylated form of RNA-Polymerase II (RNA-Pol II) during a time course of stimulation (**Figure 2A & 2B**). BMMs were starved of CSF-1 for 24 hours to allow maximal upregulation of the surface CSF1R, and *Csf1r* mRNA [28]. They were then stimulated with a combination of LPS & CSF-1 to investigate the events that occur as Csf1r is acutely down-regulated. Both primary RNA levels as well as antisense RNA were measured during a time course of stimulation. These experiments confirmed that the withdrawal of CSF-1 from control cultures led to an up-regulation of *Csf1r* RNA transcription which was associated with increased RNA Pol II over the promoter. The addition of LPS & CSF-1 caused a rapid down-regulation of RNA levels within two hours (**Figure 2A**) and an even more rapid loss of initiating RNA Pol II (**Figure 2B**). Down-regulation of RNA Pol II binding and RNA levels occurred across the promoter as well as the FIRE enhancer, indicating that there was not a primary block in elongation. Low levels of active transcription were maintained for 16 – 24 hours whereas Serine 5 phosphorylated RNA-Pol II was replenished after 24 hours (**Figure 2A & 2B**).

Primary RNA levels after 20 minutes of stimulation were still high while RNA Pol II levels were already strongly reduced. Moreover, increased amounts of RNA were detected over the FIRE enhancer. We therefore considered the possibility that this increase in RNA actually derives from antisense transcripts initiated at FIRE. Detectable anti-sense transcripts found in B cells were previously shown to initiate from two separate anti-sense transcriptional start sites within FIRE [29]. One is in the vicinity of the SP1/3/ETS/Egr-2/RUNX1 conserved element and the other overlaps an AP-1 binding site. To confirm that anti-sense transcripts are produced in primary macrophages, we performed RT-PCR on mRNA from murine macrophages using three distinct primer pairs, each of which produced bands of the predicted size that were below the limit of detection in control samples, but easily detected when cells were stimulated with CSF-1 or LPS (**Figure 2C**). To confirm that the increased RNA at 20 minutes (**Figure 2A**) when RNA Pol II levels were strongly suppressed (**Figure 2B**) is due to increased antisense transcription, we measured the synthesis of FIRE antisense (as) RNA using strand-specific primers (**Figure 2D**). Our results show a large induction of asRNA after 20 minutes of LPS & CSF-1 treatment indicating that asRNA is responsible for the increase in RNA. Unidirectional antisense initiation from FIRE and the location of the major TSS in primary mouse macrophages and in human macrophages is confirmed by genome-scale 5′ RACE (CAGE) data generated by the FANTOM consortium [30]–[31]. More recent CAGE data, sequenced at greater depth, confirms that FIRE is a unidirectional “broad” promoter, which unlike a typical TATA-less promoter, initiates transcription at multiple sites within a broad window [31]. Although there are two major TSS peaks, numerous minor peaks of initiation are detected within the 150 bp window surrounding the major TSS (Forrest A. et al. Ms submitted).The available data indicate that, in contrast to a major subclass of enhancers [32], FIRE transcription initiation is not bidirectional.

### Combined CSF-1 and LPS Treatment Induces Transient Occupation of Sp1 and AP1 binding sites within FIRE

**Figure 3:**
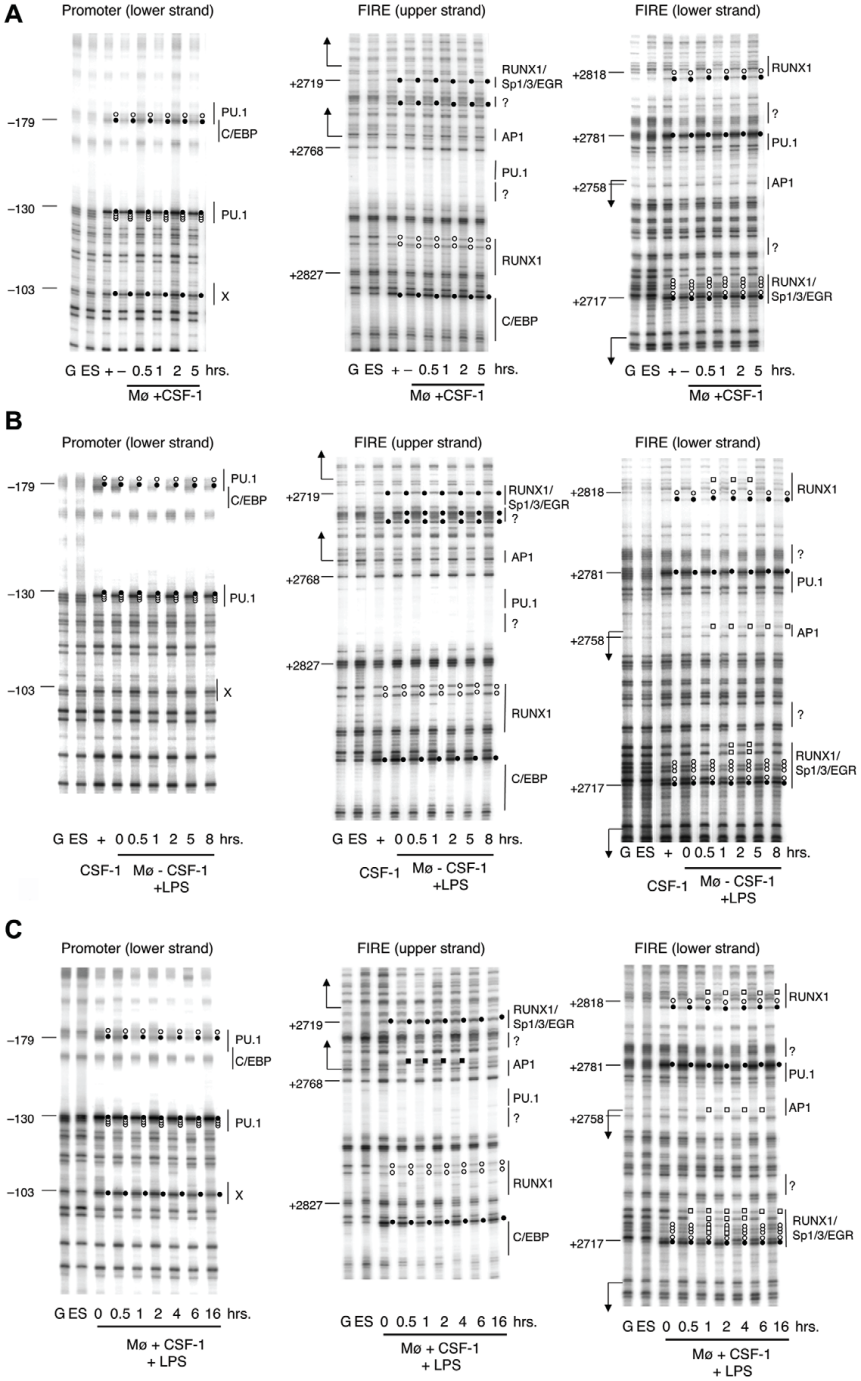
*In vivo* DMS footprinting of the *Csf1r* promoter and FIRE in stimulated BMM. Macrophages were differentiated from mouse bone marrow under the influence of CSF-1 (+) and subjected to DMS footprinting after either starving cells of CSF-1 (−) or restimulation with CSF-1 (**A**), LPS (**B**), or CSF-1 & LPS (**C**) for the indicated time points. G: Maxam-Gilbert reaction followed by LM-PCR with purified genomic DNA. ES: *In vivo* footprinting performed with ES cells. Putative transcription factor binding sites in chromatin showing alterations in methylation compared to a Maxam-Gilbert G-reaction of naked genomic DNA are shown as vertical bars on the right hand side of the gel images. Nucleotide positions relative to the ATG start are designated by numbers on the left. Macrophage specific footprints are indicated as circles (black: enhancement, white: inhibition) while LPS responsive footprints are indicated as squares (black: enhancement, white: inhibition). L-shaped arrows are the position of antisense RNA start sites at FIRE.

Transcription factor occupancy on the *Csf1r* promoter and FIRE was assessed by dimethyl sulfate (DMS) *in vivo* footprinting over a time course of treatments with CSF-1 or LPS, as indicated in **Figure 3** **A–C**. These assays indicate protein contacts at the N7 position of guanines that protect these residues from being methylated and also highlight hyper-reactivity to modification at guanines juxtaposed to such a contact [33]. Untreated macrophages displayed the characteristic and complex transcription factor binding pattern at the promoter and FIRE described previously. We observed protein-DNA contacts over binding sites for PU.1 (ETS), C/EBP, Sp and EGR family members as well as RUNX1 consensus sequences [3] and this pattern remained unchanged when the cells, after overnight growth factor withdrawal, were treated with CSF-1 alone (**Figure 3A**). LPS treatment of CSF-1 starved cells did not change transcription factor occupancy at the promoter, but resulted in weak alterations of DMS-reactivity at FIRE (**Figure 3B**). Here the LPS-induced changes were restricted to an AP1 consensus sequence and to the RUNX1/Sp1/ETS element as indicated by a reduction in DMS reactivity (white squares). These two elements are juxtaposed to the two start sites for anti-sense transcription [29]. Combined treatment with CSF-1 and LPS produced more pronounced changes (**Figure 3C**). DMS reactivity at the RUNX1/Sp1/ETS element and at the AP-1 site was reduced within 30 minutes of stimulation and returned to a pattern indistinguishable from unstimulated cells after sixteen hours. As previously described, no alterations of transcription factor occupancy were seen at the *Csf1r* promoter [3]. These data suggest that the RUNX1/Sp1/ETS and the AP-1 sites are maximally bound by protein in the presence of CSF-1 and LPS.

**Figure 4:**
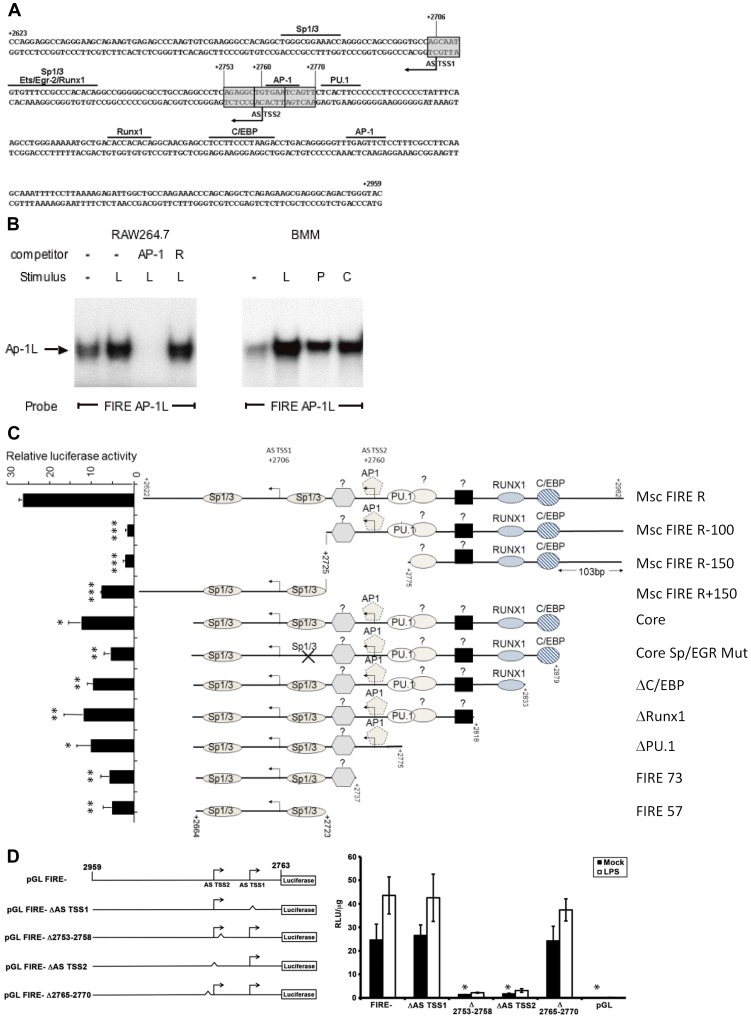
Sp1 binding sites and transcription start sites are important for promoter and anti-sense promoter activity within FIRE. (**A**) Schematic of two separate anti-sense transcriptional start sites within FIRE. One is in the vicinity of the Egr-2/RUNX1/Sp1/3 conserved element and the other overlaps the AP-1 binding site. Indicated transcription factor binding sites are based on the DMS footprinting data depicted in Figure 3. (**B**) EMSA analysis using an oligonucleotide containing the FIRE 5′ AP-1 like binding site and nuclear extract from RAW264.7 cells or BMMs with or without treatment with LPS (L), PMA (P), or CSF-1 (C). Bands were identified as AP-1 like (AP-1L) by specific competition with an oligonucleotide containing a consensus AP-1 binding site (AP-1) and not by a random sequence oligonucleotide (R). (**C**) Luciferase constructs carrying deletions of FIRE or a point mutation as indicated were transfected into RAW264.7 cells and luciferase activity was measured after 24 h. The experiments show the mean value of two independent experiments whereby each construct was transfected three times. Statistically significant differences versus Msc FIRE R are indicated (t-test; ***p<0.001, **p<0.01, and *p<0.05). (**D**) *Left*: Schematic of FIRE reverse constructs. FIRE in reverse orientation was cloned into the pGL2B (pGL2) vector upstream of a luciferase reporter (pGLFIRE-). The locations of the two antisense transcriptional start sites (AS TSS1 and AS TSS2 within context of FIRE) and the relative positions of the 6 bp deletions used in the experiment are shown. *Right*: RAW264.7 cells were transfected with each construct shown on the left or with empty pGL2B vector (pGL). Twenty-four hours post transfection, cells were treated with 100 ng/ml LPS for 6.5 h and luciferase activity was assessed. Columns represent the mean RLU/µg protein, black bars indicate mock treated samples and white bars indicate LPS treated samples. Results were almost identical in two independent experiments whereby each construct was individually electroporated three times; a representative graph is shown, with error bars representing the SEM of triplicates from a single experiment. Statistically significant differences versus mock-treated pGLFIRE- are indicated (t-test; *p<0.05).

Binding of AP-1 family proteins to the urokinase enhancer has previously been shown to be induced by CSF-1 and PMA in bone marrow-derived macrophages (BMM) [34]. Since we found that the FIRE AP-1 site was occupied in our DMS footprint analysis in cells stimulated with CSF-1 and LPS, we sought to determine if a macrophage nuclear protein complex actually bound to the site. There are two AP-1 consensus binding sites within FIRE (**Figure 4A**). The consensus for the 5′ AP-1 site (TGAATCA) and the 3′ AP-1 site (TGAGTTC) conform imperfectly to the optimal AP1 consensus (TGA[G/C]TCA). Extract from both stimulated and unstimulated RAW264.7 cells as well as BMMs were compared to determine whether inducible macrophage nuclear proteins bind to either of the putative AP-1 elements in FIRE (**Figure 4B**). EMSA of the two candidate AP-1-like elements within FIRE showed binding of an inducible protein complex that could be competed for with an oligonucleotide competitor containing the AP-1 consensus sequence from the stromelysin promoter. Both sequences showed identical protein complexes of the same relative mobility and abundance (**Figure 4B**; data not shown). Binding of the AP-1-like protein complex was inducible by LPS in RAW264.7 cells and by LPS, CSF-1 or PMA in BMMs (**Figure 4B**). These data show that binding activity that recognises the AP-1 element at FIRE is induced by agonists that repress full-length *Csf1r.* Together, the data demonstrate that maximal down-regulation of *Csf1r* transcription requires CSF-1 and LPS signalling and that these signals terminate at FIRE.

### Sp1 Binding Sites and Transcription Start Sites are Important for Promoter and Anti-sense Promoter Activity within FIRE

Two major TSS within FIRE, located +2706 bp and +2760 bp downstream of the translational ATG start site (**Figure 4A**), were shown previously to be functionally active in B cells [11], [20]. To test the function of the TSS and putative control elements in macrophages we generated a number of deletions and mutations of FIRE in a luciferase reporter and tested them in RAW264.7 cells (**Figure 4C**). The minimal promoter activity of FIRE resides on a small fragment (+2664 to +2723) containing two Sp1/3 sites which have been shown to be functional [3]. Progressive removal of other transcription factor binding sites from the 3′ end of FIRE, such as the AP-1 site overlapping the distal TSS, led to a progressive reduction in activity. Removal of the Sp1 sites by deletion of sequences upstream of +2725 severely reduced promoter activity.

We also examined six- base pair deletions spanning each anti-sense TSS (**Figure 4D** **, left**). The 5′ anti-sense transcriptional start site (AS TSS1; +2706) was dispensable for anti-sense promoter activity whereas the 3′ anti-sense start site (AS TSS2; +2760), was absolutely required. The six base pair deletion adjacent to ΔAS TSS2, Δ2753–2758 3′ in reverse orientation, were also required for anti-sense promoter activity but the six base pairs on the other side (Δ2765–2770) were not (**Figure 4D** **, right**). To confirm that the effect of deletion Δ2753–2758 on anti-sense promoter activity was not due to the distance and position relative to AS TSS1, we tested 3 other 6 bp deletions in the sequence between AS TSS1 and AS TSS2. Deletion of Δ2747–2752 or Δ2741–2746 had no effect on anti-sense promoter activity while deletion of Δ2735–2740 was required for anti-sense promoter activity. This indicates that the effect of deletion Δ2753–2758 is driven by the base pair motif and not the distance to AS TSS1 or 2 and that there are other necessary sites for anti-sense promoter activity.

### The Enhancer Activity of FIRE is Orientation Dependent and Requires the Transcription Start Sites

**Figure 5:**
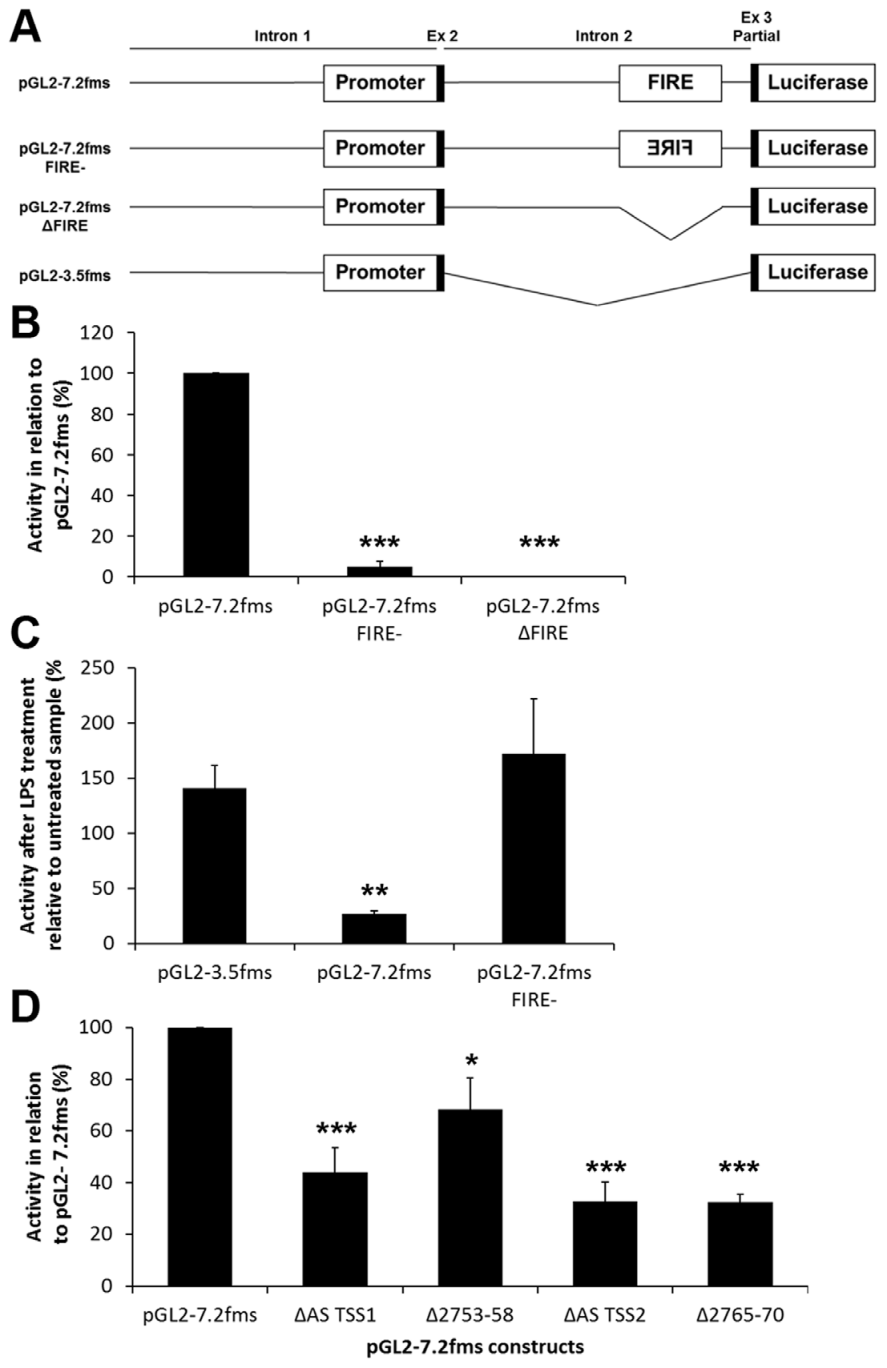
The enhancer activity of FIRE is orientation dependent and requires the transcription start sites. (**A**) Schematic of FIRE constructs: the entire *Csf1r* regulatory region plasmid (pGL-7.2 fms), pGL-7.2 fms with FIRE subcloned into the reverse orientation (pGL-7.2 fms FIRE-), pGL-7.2 fms with FIRE deleted (pGL-7.2 fms ΔFIRE), and pGL-7.2 fms with intron 2 deleted leaving 3.5 Kb of the *Csf1r* promoter (pGL2-3.5 fms). (**B**) RAW264.7 cells were transfected with pGL-7.2 fms, pGL-7.2 fms FIRE-, or pGL-7.2 fms ΔFIRE constructs and luciferase activity was assessed. Data is shown as a percentage of pGL2-7.2 fms (100%) and error bars represent the SEM. Statistically significant differences versus pGL-7.2 fms are indicated (t-test; ***p<0.001). (**C**) RAW264.7 cells were transfected with pGL2-3.5 fms, pGL-7.2 fms, or pGL-7.2 fms FIRE- constructs. Following treatment with LPS, luciferase activity was assessed. Data for each construct are shown normalised to the same construct untreated and error bars represent the SEM. Statistically significant differences between the LPS treated construct versus the same construct untreated are indicated (t-test; **p<0.01). (**D**) RAW264.7 cells were transfected with linearized pGL-7.2 fms or a linearized pGL-7.2 fms construct containing one of the four 6 bp deletions in FIRE shown in Figure 4D. Forty-eight hours post transfection, luciferase activity was assessed. The data represents six separate experiments performed in triplicate and error bars represent the SEM. Data are shown as a percentage of pGL2-7.2 fms (100%). Statistically significant differences versus pGL-7.2 fms are indicated (t-test; *** p<0.001, ** p<0.01, and * p<0.05).

FIRE enhancer activity is essential for basal *Csf1r* transcription so dissection of the role of antisense transcription by mutational analysis in mice is not straightforward. The traditional view of enhancers is that their function is orientation-independent. To test this, we produced a luciferase reporter construct in which the orientation of FIRE was reversed without introducing any other sequence changes. Luciferase reporter constructs containing the 3.5 kb promoter alone (pGL2-3.5 fms), the intron-containing native 7.2 Kb construct (pGL2-7.2 fms), or constructs in which the 300 bp FIRE region was removed (pGL2-7.2 fms ΔFIRE) or inverted (pGL2-7.2 fms FIRE-) were tested (**Figure 5A, B & C**). As reported previously [7], the inclusion of the intron (pGL2-7.2 fms) reduced the reporter gene expression in RAW264.7 cells compared to the 3.5 kb promoter alone (pGL2-3.5 fms; data not shown). As observed with EGFP-reporter genes in transgenic mice [8], but not previously studied in transient transfections of RAW264.7 cells, a construct in which FIRE was removed (pGL2-7.2 fms ΔFIRE) was completely inactive. Replacement of FIRE in the reverse orientation (pGL2-7.2 fms FIRE-) restored less than ten percent the activity of the reporter plasmid (**Figure 5B**). Additionally, the reporter constructs were treated with LPS to determine the effect on activity. The pGL2-3.5 fms plasmid was LPS unresponsive, whereas the activity of pGL2-7.2 fms was repressed by LPS. The pGL2-7.2 fms FIRE- actually had increased activity in the presence of LPS demonstrating that reversing enhancer orientation prevented inhibition by LPS (**Figure 5C**). To confirm the role of antisense transcription in the enhancer activity of FIRE, we inserted the same 6 bp deletions tested in **Figure 4D** into the 7.2 kb intron-containing reporter construct (pGL2-7.2 fms). Mutation of either of the TSS caused a partial reduction of reporter gene expression. The six base pair deletion adjacent to ΔAS TSS2, Δ2765–2770, were also required for enhancer activity but the six base pairs on the other side (Δ2753–2758) were not (**Figure 5D**).

## Discussion

### Antisense Promoter Activity Correlates with the Regulation of *Csf1r* Expression

FIRE is a complex enhancer element that is essential for macrophage-specific expression of the *Csf1r* gene, but at the same time also required for acute regulation by a range of stimuli. The dissection of these different properties is a difficult task. The Ets family transcription factors, PU.1 and Ets2 transactivate the *Csf1r* promoter and the purine-rich target elements bound by these factors are necessary and sufficient for transcription initiation in macrophages. LPS, CSF-1 and PMA regulate both PU.1 and Ets2 at their level of expression and phosphorylation [5], [35], [36]. However, this did not impact on their activity on the *Csf1r* promoter as its basal activity was marginally activated by agents that repress mRNA expression (**Figure 1**). Instead, our data show that altered promoter activity is a consequence of altered transcription factor binding to FIRE. Stimulation with LPS and CSF-1 altered occupancy of functional binding sites for the transcription factors AP-1 and Sp1 (**Figure 3**), correlating with increased abundance of these factors in the nucleus. This indicates that FIRE enhancer activity and FIRE promoter activity are driven by alternative sets of transcription factor complexes. Activation of antisense transcription with LPS, PMA or CSF-1 correlates with a decrease in expression. This finding recapitulates the observation in B cells that PAX5 acts to repress *Csf1r* transcription by blocking the main promoter but also inducing the antisense promoter [11]. The enhancer activity of FIRE is associated with the deposition of active histone marks likely to require PU.1, C/EBP and RUNX1 (AML1), all of which bind to FIRE in both mouse and human. The latter protein binds to two sites within mouse FIRE, and expression of *Runx1* mRNA and nuclear binding activity is repressed by CSF-1 [28] which would be consistent with the up-regulation of *Csf1r* mRNA in the absence of CSF-1(**Figure 4A**).

We previously provided evidence that the *Csf1r* proximal promoter was active in non-macrophage tumour cells, and was growth factor-responsive [23]. We suggested that FIRE acted in part to prevent read through of the intron, producing a block to transcription elongation, and that this might also explain the repression by LPS, PMA and CSF-1 [21]. However, this mechanism of regulation is incompatible with the data in **Figure 2**. Instead, the data demonstrates a complex interplay between the main promoter and FIRE in response to inflammatory stimuli which leads to direct and rapid repression of transcription initiation. That conclusion is consistent with earlier studies of LPS action using nuclear run-on transcription [37]. CSF-1 starvation of BMM produced an increase in primary *Csf1r* transcripts from the promoter and also from FIRE, paralleled by increased association of active RNA Pol II. Addition of LPS and CSF-1 caused an immediate increase of the antisense transcripts followed by a rapid decrease of active RNA Pol II occupancy and a slow decrease in primary transcripts across the coding region. This precise temporal correlation suggests that the interplay between sense and antisense transcription is of relevance for the regulation of *Csf1r* mRNA levels. However, while the effects of transcriptional interference have been described in yeast [38] there have been few studies of the consequences of bidirectional transcription initiation in mammals and more elaborate experimentation is necessary to dissect the precise molecular details at *Csf1r*.

### The Activity of FIRE *in situ* is Orientation Dependent

Enhancers are supposed to act in a position and orientation-independent manner. However, reversing an enhancer in its native context has rarely formally tested this definition. Our experiments clearly demonstrate that in its native context the enhancer activity of FIRE is orientation dependent. Experiments with stably transfected cells and transgenic mice have shown that the *Csf1r* promoter alone is insufficient to mediate maximal expression of a reporter gene in a chromatin environment. Moreover, in the absence of FIRE, the presence of intronic sequences repressed this basal activity even further [7]. FIRE may therefore act as an anti-repressive element, relieving transcription arrest by so far uncharacterised repressive elements within the intron. This essential “anti-repressive” activity is ablated by reversing the orientation of FIRE (**Figure 5B**). By inference, the antisense transcript may contribute to the ability of the FIRE sequence to overcome repression by the remainder of the intron. A similar mechanism has been documented in the LPS-induced up-regulation of the lysozyme locus [39]. Here transcription from an inducible antisense promoter upstream of the normal transcription start site suppresses the activity of a silencer element by reorganising chromatin architecture and altering transcription factor binding. Cook et al. [40] first suggested that transcription arising from enhancers may act to focus enhancers and target promoters into active transcription factories, facilitating cis-element interaction and concentrating transcriptional regulators around the start site. The data herein could support such a mechanism of action for FIRE. A cis-acting mechanism is also favoured by a lack of trans-acting effects of the intron. Co-transfection of a 10-fold excess of the 7.2 kb *Csf1r* promoter fragment containing FIRE, had no effect on expression of the 7.2 kb *Csf1r-*luciferase reporter gene in RAW264.7 cells (data not shown). The fact that many enhancers generate transcripts makes it likely that the activity of such elements to stimulate transcription in their native chromatin environment could also be orientation dependent.

## Materials and Methods

### Ethics Statement

Animals were allowed free access to food and water and were maintained under temperature, humidity and light-controlled conditions. In accordance with the United Kingdom Animal (Scientific Procedures) Act of 1986, this study did not require a Home Office project license because no regulated procedures were carried out. Mice were humanely killed at a designated establishment by exposure to carbon dioxide gas and/or dislocation of the neck, which are appropriate methods under Schedule 1 of the Act.

### Cell Culture

Bone marrow cells were isolated from the femurs and/or tibias of adult C57Bl/6 mice exactly as in Schroder, et al. [17] and cultured in RPMI or IMDM media containing penicillin-streptomycin, GlutaMAX-I supplement, and 10% fetal bovine serum. Cells were differentiated to macrophages by a 6–7 day treatment with 1000 units of human CSF-1 (Chiron Corp., Emeryville, CA) or with 10% L929 conditioned medium as a source of mouse CSF-1. RAW264.7 cells (obtained from the American Type Culture Collection) were cultured in RPMI medium with the above supplements.

### Plasmids

The pGL0.5 fms, pGLFIRE-, pGL-7.2 fms, pGL-3.5 fms, pGL-7.2 fms ΔFIRE plasmids have been described previously [7]. All reporter constructs in **Figure 4C** carrying deletions or point mutations within FIRE have been described previously [11]. The pGL-7.2 fms FIRE- plasmid was produced by deletion of the FIRE region using splice overlap PCR on an Xho1 fragment at the end of intron 2 excised from the pGL-7.2 fms plasmid. Primers for splice overlap introduced an Asc1 site into the sequence and FIRE was cloned in the inverted orientation before reinsertion of the Xho1 fragment into the pGL-7.2 fms plasmid. In **Figure 4D**, the generation of pGLFIRE- six base pair (bp) deletion mutants were generated across the 337 bp FIRE sequence using splice overlap PCR using a modified protocol based on published reports [18], [19]. Universal external PCR primers were designed with flanking AscI sites to facilitate cloning. TAGC AscFire CB F: 5′TACGGGCGCGCCCCAGGAGGCCAGGGAAGC3′ TACG FIRE CB ASC R: 5′TACGGGCGCGCCGTACCCAGTCTGCCCTCG3′ Internal primers containing six bp deletions were systematically designed across FIRE with eighteen bp flanking each side of the deletion. PCR reactions contained a final concentration: 1 mM MgSO4, 0.3 mM each dNTP, 0.4 mM each primer and 0.5U Platinum Pfx Polymerase (Invitrogen, Carlsbad, IN). Cycling parameters were 94°C for 2 min; 94°C for 15 sec, 60°C for 30 sec, 68°C for 30 sec for 30 cycles followed by 68°C for 1 minute once. PCR products were gel purified using Qiagen Extraction Gel Kit (QIAGEN, Valencia, CA) as per manufacturer’s instructions. Two microliters of each PCR reaction for each mutation was used for second round PCR. Second round PCRs were performed and cycled as above but no primers were added and fifteen cycles were performed. After fifteen cycles, the external primers (TAGC AscFire F2 and Asc Univ Fire Rev) and 0.2U Taq were added and cycled for 30 rounds of PCR. PCR products were purified using Qiagen Gel Extraction Kit, as above and then digested with AscI (NEB), and heat inactivated for 20 min at 65°C. These products were directly ligated into PGL2B that had an AscI site inserted into the SmaI site to facilitate cloning purposes. Correct clones were confirmed by AscI digest and sequence analysis. In **Figure 5D**, the generation of pGL-7.2 fms six base pair (bp) deletion mutants were generated using splice overlap PCR using a modified protocol based on published reports [18], [19]. Universal external PCR primers were designed within FIRE as well as internal primers containing the same four 6 bp deletions from **Figure 4D** with eighteen bp flanking each side of the deletion. PCR conditions were the same as above. These products were directly ligated into pGL-7.2 fms using native XhoI sites. Correct clones were confirmed by restriction enzyme digest and sequence analysis.

### Transfection

Stable transfections were performed by electroporation of 5×10^6^ cells in 250 µl RPMI media containing 20 mM HEPES and 10 µg reporter with 1 µg pPNT neomycin resistance plasmid at 280 volts and 1000 µFarad capacitance on a Bio Rad Gene Pulser (Bio Rad), followed by selection with 250 µg/ml Geneticin. For stable transfection, antibiotic resistant cells with or without 48 hours of LPS treatment were analysed on a Facstar flow cytometer (Becton Dickinson). The extended treatment with LPS was required to see repression of the stable EGFP protein. Transient transfections were performed as above with 10 µg of reporter plasmid without the additional antibiotic resistance plasmid. In **Figure 4C**, RAW264.7 cells were transfected using a 1∶3 ratio of lipofectamine and plasmid (0.6 µg of FIRE constructs reporter vectors, 0.25 ng of Renilla and 0.2 µg of pBluescript) in 250 µL Opti-mem (Gibco; Invitrogen). Cells were transfected for 24 hours.

### Treatment

**Figure** **1**: (B) 24 hours post-transfection, cells were stimulated with either 20 ng/ml LPS or DNA for 8 hours before lysis. (C) 24 hours post-transfection, cells were stimulated with either 1000 U/ml of CSF-1 for 20 hours, PMA (10^−7^ M) for 8 hours, or a combination of CSF-1 and PMA before lysis. **Figure** **2** (A/B/D): Cells were cultured in M-CSF free medium for 24 hours and subsequently treated with 20 ng/ml LPS (Sigma) and 50 ng/ml murine recombinant M-CSF (Peprotech) for 20 min to 24 hours. (C): Cells were stimulated with either 20 ng/ml LPS or 1000 U/ml of CSF-1 for 8 hours. **Figure 4** (D): 24 hours post-transfection, cells were stimulated with 100 ng/ml LPS for 6.5 hours **Figure 5** (C): 24 hours post-transfection, cells were stimulated with 100 ng/ml LPS for 8 hours.

### Luciferase Assay

Luciferase activity was assayed according to the manufacturer’s protocol (Roche Biochemical). pGL2 basic and pGL2 luciferase reporter vectors were used as controls. The concentration of protein was determined by BCA protein assay (Pierce) and the level of luciferase activity was given as relative light units (RLU), calculated as light units/ µg of protein assayed. Samples in **Figure 4**C were assayed for luciferase activity using the Promega Dual Luciferase assay system.

### RNA Isolation and Quantification

Total RNA was prepared using RNeasy isolation kits (QIAGEN) according to the manufacturer’s instructions or by TRIzol® (Invitrogen). RNA was treated with DNaseI and reverse transcribed into cDNA using reverse transcriptase. For **Figure 2A**, cDNA were quantified using real-time quantitative polymerase chain reaction (qPCR) with SYBR Green, ABI 7500 real-time PCR system (Applied Biosystem), and the following oligonucleotides: c-fms prom (For: CTGCTGCTGGCCACAGTTT and Rev: CAGCGATGCCCTCTTTGC), Fire 5′ (For: GGAAACCCTGAAGTCCTCTAAGG and Rev: TGACCCCGCAAGTCAACC), FIRE 3′ (For: CAGACTGGGTACCTCTCTCCTTCAT and Rev: GCCAGGAAACCGTTGTGTCAT) and intron 2 (For: TGACCAAGCACCTAGAGCAA and Rev: GATCAAAGGCCTGAGCATCT). For **Figure 2C**, negative control samples (no first strand synthesis) were prepared by performing reverse transcription reactions in the absence of reverse transcriptase. Detection of antisense RNA was determined by priming of mRNA with the following oligonucleotides: ASp1; GGAGAAGTGTCTGGAACC, ASp2; CTGGCTGGTTGGAGGCTTGG and PCR amplification with NF1; GTACGGCCTGAGAACAGCAGGTGG, NF2; GAGTTTGAATCAGAGTGA, NF3; CAGGACAGTGGCACAGCACAGACA NR; TGTCCTTCTCACACACTGAGGGAC.

### Antisense RNA Assay

Antisense RNA-expression was assayed exactly as described in [20]. Briefly, cDNA was synthesised from 2 µg of DNaseI-treated total RNA by using 400 U of M-MLV reverse transcriptase and biotinylated primers specific for antisense-*Csf1r* transcript ([Btn]GGTCAGCAAACAGGACAGTGGCACAGCACAGACAG) (2 pmol) and rDNA ([Btn]GCACCGCGACAGACCCAAGCCAGTA) (0.2 pmol) in one reaction. Synthesised cDNA was immobilised on Dynabeads (Dynal, M-280). RNA and trace amounts of genomic DNA were removed by alkaline denaturation and serial washing. cDNA was eluted by heating the bead suspension in 0.1TE for 15 min at 95°C and was followed by qPCR using antisense-*Csf1r* (FIRE4 for: TGTGCCCAGTCTGCTCTAA and FIRE4 rev: CTCCTGGCCATTGTCCTTCTC) and rDNA (rDNA for: CTGTCCTTTCCCTATTAACACT and rDNA rev: GAATAGGCTGGACAAGCAAAACA) primers. Primers amplification efficiency was calculated using genomic DNA as a standard. Antisense-*Csf1r* expression was normalised against rDNA signals.

### Chromatin Immunoprecipitation Experiments

Sub-confluent bone marrow derived macrophages were harvested and washed in cold PBS. Chromatin was cross-linked with 1% formaldehyde (Thermo Fisher Scientific, IL USA) for 1 hour at 4°C. The cells were lysed in 10 mM HEPES (pH 8), 10 mM EDTA, 0.5 mM EGTA, 0.25% Triton X100 and protease inhibitor cocktail (PIC). The nuclei were collected by centrifugation and lysed in 10 mM HEPES (pH 8), 200 mM NaCl, 1 mM EDTA, 0.5 mM EGTA, 0.01% Triton X100 and PIC. Chromatin was transferred to IP buffer containing 25 mM Tris-HCl (pH 8), 2 mM EDTA, 150 mM NaCl, 1% Triton X100, 0.25% SDS, PIC and sheared using a Bioruptor sonicating water bath (Diagenode) to an average length of 200–500 bp. The chromatin solution was diluted in 25 mM Tris-HCl (pH 8), 2 mM EDTA, 150 mM NaCl, 1% Triton X100, 7.5% glycerol and antibody precipitations were performed. Chromatin from 10^6^ cells and 10 µl of Dynabeads protein G (Invitrogen) coupled with 1 µg of RNAP II S5 antibody (ab5131; Abcam, Cambridge, United Kingdom) were incubated for 2 hours at 4°C with rotation. The immune complexes were collected using a magnet separator and washed with low salt wash buffer 1 (20 mM Tris-HCl (pH 8), 2 mM EDTA, 1% Triton X100, 0.1% SDS, 150 mM NaCl), high salt wash buffer 2 (20 mM Tris-HCl (pH 8), 2 mM EDTA, 1% Triton X100, 0.1% SDS, 500 mM NaCl), LiCl buffer (10 mM Tris-HCl (pH 8), 1 mM EDTA, 0.25 M LiCl, 0.5% NP40, 0.5% Na deoxycholate) and TE/NaCl buffer (10 mM Tris-HCl (pH 8), 1 mM EDTA, 50 mM NaCl). Precipitated DNA was eluted in 1% SDS and 100 mM NaHCO_3_ and followed by crosslink reversal by heating at 65°C overnight. Chromatin DNA was purified using Ampure PCR purification kit (Agencourt AMPure, APN 000130) and quantified using real-time qPCR with SYBR Green. Primers used in this assay were Prom -1.5 kb (For: CACGCCGGCTGAGTGTCT and Rev: TCCACGTAGATGGTGTCAGCAT), c-fms prom (For: CTGCTGCTGGCCACAGTTT and Rev: CAGCGATGCCCTCTTTGC), Fire 5′ (For: GGAAACCCTGAAGTCCTCTAAGG and Rev: TGACCCCGCAAGTCAACC), FIRE 3′ (For: CAGACTGGGTACCTCTCTCCTTCAT and Rev: GCCAGGAAACCGTTGTGTCAT) and intron 2 (For: TGACCAAGCACCTAGAGCAA and Rev: GATCAAAGGCCTGAGCATCT).

### *In vivo* DMS Footprinting

Bone marrow macrophages (BMM) were deprived of CSF-1 for 24 hours, and then restimulated with CSF-1 (10% L929 cell conditioned medium) or 20 ng/ml LPS or the combination as indicated. At the end of each incubation time, cells were washed with PBS and treated with 0.2% DMS/PBS for 5 minutes at room temperature. After three washes with ice-cold PBS, genomic DNA was extracted from cells and subjected to piperidine cleavage and Ligation-mediated PCR (LM-PCR) amplification. LM-PCR was performed as described previously [3].

### EMSA

Isolation of nuclei and extraction of protein from BMMs and RAW264.7 cells was performed according to established protocols and the concentration of protein in nuclear extracts was determined by BCA protein assay. The double stranded oligonucleotide probes used contained the following sequences; CSF-1R Sp1: CAGGCTGGGCGGAAACCA CSF-1R Ap-1: AGGCTGTGAATCAGTTCTCA. Binding reactions contained 20 mM HEPES buffer pH 7.4, 40 mM NaCl, 15% glycerol, 1 mg of protein from nuclear extract, 25 ng poly (dI-dC) and approximately 0.01 pmole of ^32^P labelled probe for 20 minutes at room temperature. Antibody supershifts were performed by pre-incubating binding reactions with α-Sp1 antibody (Santa Cruz Biotechnology) for 15 minutes at 4°C before addition of probe and protein binding. Competitions were performed by addition of unlabelled oligonucleotide corresponding to the stromelysin AP-1 consensus element at a ratio of 20 to 1 immediately before addition of labelled probe. Protein binding was resolved on 5% mini-polyacrylamide gels (Bio Rad) in 0.5 XTBE (45 mM Tris base, 45 mM boric acid, and 1 mM EDTA) for 1 hour at 100 volts.
